# A study on the equilibrium of older adult care service resources and demand in Guangzhou

**DOI:** 10.3389/fpubh.2026.1775866

**Published:** 2026-02-26

**Authors:** Lianhua Liu, Yakang Liu, Shiqi Lyu, Lifen Zheng

**Affiliations:** 1 School of Management, Guangzhou Huashang College, Guangzhou, Guangdong, China; 2 Faculty of Humanities and Social Sciences, Macao Polytechnic University, Macau, China; 3 Geisel School of Medicine, Dartmouth College, Hanover, NH, United States

**Keywords:** older adult care service demand, older adult care service resource, older adult care service system, equilibrium, population aging

## Abstract

**Introduction:**

Population ageing in Guangzhou has continued to intensify, and the older adult population is increasingly concentrated in central urban areas. This concentration amplifies spatial mismatch between service demand and resource allocation, thereby shaping both the equity of public service provision and the capacity of urban governance.

**Methods:**

Using district-level data on older adult care service demand and care-service resources in Guangzhou from 2015 to 2023, we develop an integrated analytical framework combining the entropy-weight method, spatial autocorrelation analysis, geographic concentration measures, and inconsistency indices. This framework is applied to identify the spatial structure of demand–resource alignment and its evolution over time.

**Results:**

The analysis reveals a clear core–periphery pattern. Central urban districts form structural “bottleneck zones,” characterised by high service demand and insufficient resource supply. Peripheral districts, by contrast, display a reverse mismatch: comparatively higher pre-allocated resources alongside lagging or slower-growing demand. Transitional districts between the urban core and outer areas exhibit relatively better coordination between demand and resources.

**Discussion:**

Overall, Guangzhou’s older adult care system appears to be entering a phase of structural adjustment in which misalignment is driven less by absolute scarcity alone and more by spatial configuration and cross-district spillovers. Policy priorities should therefore shift toward spatial optimisation of facilities and service capacity, strengthened cross-district coordination mechanisms, and more targeted resource allocation that responds to the evolving geography of the older population and care needs.

## 1 Introduction

As a national central city and a core engine of the Guangdong–Hong Kong–Macao Greater Bay Area, Guangzhou has long maintained robust economic vitality and a relatively well-developed public service system. However, the sustained transformation of its population structure is driving profound changes in the logic of urban governance. Recent official statistics indicate that the proportion of the older population in Guangzhou has exceeded 18%. At the same time, aging levels in central districts such as Yuexiu, Liwan, and Hai Zhu have surpassed 20%, marking the city’s steady entry into a stage of deep population aging. Aging is no longer merely a matter of population size but has increasingly manifested as a structural and spatial phenomenon. The growing geographic mismatch between concentrations of the older population and the provision of healthcare, older adult care, and community-based services has led to declining efficiency in public resource allocation, while simultaneously intensifying pressures on grassroots governance and fiscal support systems. These dynamics have emerged as critical constraints on the city’s sustainable development.

The spatial distribution of the older population has gradually shifted from a pattern driven primarily by natural demographic growth to a composite structure shaped by public service accessibility, the density of medical facilities, and the coverage of social security systems. Existing studies have largely focused on longitudinal descriptions of aging trends or macro-level policy responses, with relatively limited attention to systematic analytical frameworks centered on spatial measurement and resource allocation. As a result, the spatial mechanisms underlying supply–demand mismatches and their implications for urban governance have not been sufficiently examined. Against the backdrop of increasingly refined demands for population governance, it is necessary to develop a data-driven spatial-matching framework to support institutional adjustments in public resource planning, older adult care facility layout, and cross-district coordination mechanisms. This study aims to systematically identify the spatiotemporal coupling structure between population aging and older adult care resources in Guangzhou, thereby providing empirical evidence and policy insights for establishing a resource allocation system that balances equity and efficiency.

## 2 Literature review

### 2.1 Spatial patterns and evolution of population aging

Population aging is not merely the outcome of demographic structural change but a complex spatiotemporal phenomenon embedded in regional development, population mobility, and processes of spatial restructuring. Since the beginning of the twenty-first century, population aging in China has evolved simultaneously in terms of pace, depth, and heterogeneity, exhibiting pronounced spatial differentiation (1–10). Using provincial-level cross-sectional data from 2002 to 2018, Wang et al. (1) found that the center of population aging has gradually shifted toward northeastern China, while the economic center has moved in the opposite direction toward southwestern regions. This divergence has resulted in a clear spatial mismatch between population aging and economic development.

Existing research has progressively shifted from provincial and municipal scales to finer spatial units, including subdistricts, townships, and grid-level analyses. At the subdistrict and township scale in the Yangtze River Delta, Xu et al. (2) demonstrated that aging rates in suburban areas exceeded those in central urban districts by approximately six percentage points. Net population outmigration was identified as the primary driver of this pattern. The urban–rural dual structure has been widely recognized as a key mechanism shaping the spatial configuration of population aging in China. Chan et al. (3) reported that in 2020, the proportion of individuals aged 65 and above in rural areas reached 17.7 percent, exceeding the urban level by 6.6 percentage points. A pronounced aging gradient was observed across the two sides of the Hu Huanyong Line (3).

From a methodological perspective, Shiode et al. (4) argued that a single indicator, such as the aging rate, is insufficient for identifying latent concentrations of older populations within cities. They proposed the combined use of aging density and aging proportion to better capture differentiated spatial patterns, including high-concentration older areas in central cities and aging belts in rural regions affected by population hollowing. This analytical approach has since been widely adopted in studies of Chinese megacities (6). Overall, existing studies suggest that the spatial process of population aging in China does not follow a pattern of homogeneous diffusion. Instead, it exhibits a complex structure characterized by regional differentiation between the northeastern and southwestern areas, persistent urban–rural disparities, and the coexistence of outward and inward redistribution processes within cities.

Research on the spatial equilibrium of older adult care services in international megacities has established a relatively stable theoretical and methodological framework. Within the framework of local public goods provision and jurisdictional competition, the Tiebout model (7) provides a foundational mechanism for explaining spatial variation in the allocation of quasi-public services such as eldercare. Within the tradition of spatial equity measurement, Talen (8) established the analytical logic of “facility distribution—group disparities—equity assessment” by focusing on service accessibility and social fairness. As aging deepens, international metropolitan experiences increasingly emphasize the “embeddedness in daily life” of care infrastructure. For instance, Ujikawa’s (9) Tokyo study reveals that everyday commercial nodes, such as convenience stores, can function as quasi-care infrastructure within crisis-driven care landscapes, reflecting micro-level spatial reorganization mechanisms in urban care systems. In high-density urban contexts, Zhang et al. (10) revealed the structurally fluctuating nature of London’s older adult health service accessibility based on temporal dynamics and transportation conditions, highlighting the interplay between time and service system rhythms. Wong et al. (11), examining U.S. long-term care facilities, approached the issue through spatial-ethnic inequalities, demonstrating significant spatial clustering and structural disparities in pandemic risk and resource vulnerability among care institutions. Collectively, these studies indicate that spatial mismatches in eldercare services represent not merely static deviations between “resources and demand,” but rather form an explanatory loop involving local governance structures, facility accessibility mechanisms, and the spatial distribution of vulnerable populations. Accordingly, this paper’s measurement and discussion of spatial equilibrium between eldercare service demand and resources within the Guangzhou context can foster a comparable theoretical dialogue with existing research on spatial equilibrium in eldercare services across international megacities.

### 2.2 Current research on the equilibrium of older adult care service demand and resources

#### 2.2.1 Research on older adult care service demand

Research on older adult care service demand has gradually shifted from a single-dimensional focus on age structure to a multidimensional framework integrating health status, behavioral characteristics, and environmental conditions. Measurement of older adult care service demand has increasingly shown tendencies toward spatialization, micro-level analysis, and scenario-based assessment. These approaches emphasize individual health conditions, behavioral preferences, and spatial environments as core variables, aiming to construct community-level demand maps that can be directly applied to public service planning (6, 12–16).

Zhang et al. (6), in their study of older adult care service demand in Guangzhou, found that accessibility to parks, hospitals, and supermarkets contributed 0.82, 0.68, and 0.24, respectively, to positive emotional well-being among older adults. In contrast, high population density was shown to increase negative emotional outcomes significantly. Using national data from the Chinese Longitudinal Healthy Longevity Survey, Li and Liu (12) demonstrated that each one-level increase in the accessibility of community health services was associated with a 0.22-point increase in Activities of Daily Living scores among older adults. The magnitude of this effect in urban areas was approximately twice that observed in rural areas (12). Zhang et al. (17) constructed a potential demand index by integrating disability rates, chronic disease prevalence, and willingness to enter institutional care. Their projections indicated that by 2030, approximately 32 percent of older adults in Shanghai would have demand for institutional older adult care services, far exceeding the number of beds planned under existing policy frameworks (13). From a migration perspective, recent studies have further differentiated older adult care service demand into distinct types. Li et al. (14) classified older population mobility into relocation for retirement and migration accompanying family caregiving. Their findings showed that the former was primarily concentrated among retired populations from northeastern and northern China, while the latter was highly clustered in coastal provincial capital cities. These two mobility patterns were associated with markedly different structures of public service demand, thereby contributing to spatial disequilibrium in older adult care service demand across regions (14).

#### 2.2.2 Research on older adult care service resource provision

Supply-side research on older adult care services has primarily focused on how much to allocate, where to allocate, and how effectively resources are allocated, gradually forming an integrated analytical framework encompassing accessibility, equity, and efficiency (17–30). Within this framework, spatial accessibility has been widely adopted as a core indicator for evaluating the equilibrium of older adult care service resources. (21) assessed the comprehensive accessibility of six categories of community-based older adult care facilities in the inner city of Shanghai. Their results showed that Huangpu District had the highest accessibility within a 15-min living circle, whereas extensive service-blind areas were identified in six peripheral districts. Geographically weighted regression analysis further revealed a negative association between accessibility and the density of medical and catering facilities, suggesting that excessive spatial concentration may inhibit effective service coverage (18). Using a dynamic evaluation based on the enhanced two-step floating catchment area method, Sun et al. (19) examined institutional bed provision in Beijing from 2010 to 2020. Although the total number of beds increased by 72 percent during this period, accessibility in ecological conservation areas declined by 18 percent. Meanwhile, the concentration of beds in central urban areas and newly developed towns resulted in an average utilization rate of only 61 percent, indicating pronounced spatial disequilibrium in resource allocation (19).

Equity has also been a major focus of supply-side studies. Zhang et al. (17) decomposed the Gini coefficient of older adult care service resources at the provincial level and found that 70 percent of overall disparities originated from intraregional inequality. The degree of inequality within western provinces was approximately 1.8 times higher than that observed in eastern provinces (17). In terms of efficiency evaluation, recent studies have increasingly applied the data envelopment analysis slack-based measure model. Zhang et al. (20) estimated provincial-level allocation efficiency in 2020 and reported that 77 percent of provinces operated on the efficiency frontier. However, most western provinces were located in a quadrant characterized by low supply and low utilization, highlighting a pronounced spatial trade-off between equity and efficiency in the allocation of older adult care service resources (20).

Overall, existing supply-side research indicates that older adult care service resource allocation in China has shifted from overall insufficiency to one dominated by structural disequilibrium. These findings underscore the urgent need for more refined, community-oriented spatial planning strategies to improve the equilibrium of older adult care service resources.

#### 2.2.3 Advances in research on the equilibrium between older adult care service resources and demand

Research on the equilibrium between older adult care service resources and demand has adopted multiple methodological approaches, including coupling coordination analysis, spatial disequilibrium indices, and multi-agent simulation models. These studies consistently reveal a set of structural contradictions characterized by overall quantitative balance, spatial disequilibrium, and mismatches across service types (13, 31–35). Such findings indicate that apparent aggregate adequacy may conceal pronounced spatial and structural inefficiencies in older adult care service systems.

Using Gansu Province as a case study, Liu et al. (31) constructed a coupling coordination model linking population aging and the number of institutional care beds. Their results showed that in 2020, the provincial coupling degree was 0.42, indicating a low level of coordination. Further analysis using a geographic detector model indicated that the interaction between population density and fiscal investment explained 0.75 of the observed spatial variation. However, economically disadvantaged areas in the north simultaneously faced high levels of population aging and insufficient resource provision, reflecting a persistent spatial disequilibrium (31). Zhu et al. (2022) applied a multi-agent simulation approach in Shanghai and projected that by 2030, the number of institutional care beds could reach 158,000 under existing planning schemes. Despite this increase, spatial disequilibrium would persist, with central urban districts such as Xuhui, Jing’an, and Putuo still facing a combined shortage of approximately 19,000 beds. In contrast, some newly developed suburban towns were projected to experience utilization rates below 40 percent (13). At a finer spatial scale, Jin et al. (32) employed the generalized two-step floating catchment area method in Jinan. They found that small-scale older adult care institutions exhibited the greatest disparities in spatial accessibility. Accessibility levels in high-demand subdistricts were only one-third of those observed in low-demand areas, resulting in an inverted structure characterized by high demand and weak service provision (32). Collectively, these studies indicate that in large cities, the core contradiction in older adult care service provision has shifted from availability to appropriate allocation. Spatial disequilibrium between older adult care service resources and demand has thus emerged as a critical bottleneck constraining improvements in system performance and public service effectiveness.

In research on balanced public services, the core concept of geographic concentration is comparing “the share of a given variable within spatial units” with “spatial benchmark shares,” often measured by land area or population share, to quantify deviations. The “geographic concentration index” systematically presents this concept and its operational form, providing a standard reference for cross-regional and cross-scale comparative measurement. Building upon this framework, recent spatial consistency studies typically define the ratio of concentration levels between different factors—such as population-economy or demand–supply—as an “inconsistency index.” This index characterizes the relative advancement or lag in spatial agglomeration intensity between two factors, thereby elevating “concentration measurement” to “matching relationship assessment” (36). In supply–demand matching applications, Liu et al. (31) noted that ratio-based inconsistency indicators derived from concentration measures have been employed to identify structural deviations between resource allocation and demand agglomeration. Regions are then categorized into types such as “supply-leading,” “coordinated,” and “supply-lagging” based on thresholds, yielding verifiable typologies with interpretable policy implications (37). In summary, the adopted geographic concentration and mismatch indices possess clear theoretical origins and cross-study comparability, aligning with the public service equilibrium measurement paradigm within the aforementioned methodological spectrum: the former focuses on the spatial agglomeration intensity of a single factor, while the latter utilizes the ratio of two factors’ concentrations to achieve directional identification of supply–demand matching.

### 2.3 Progress in research on older adult care services and resource allocation in Guangzhou under the context of integrated older adult care in the Greater Bay Area

Research on Older Adults Care Services in the Guangdong-Hong Kong-Macao Greater Bay Area focuses on “Reconfiguring Cross-City Older Adults Care Demand—Smart Supply Spatial Reallocation.” Xiang and He (38) examined the demand side of older adult care in the Guangdong-Hong Kong-Macao Greater Bay Area, noting that Hong Kong residents’ perceptions of mainland healthcare quality significantly shape their migration and cross-regional retirement intentions, leading to cross-city redistribution of older adult care demand. (6) argued that differences in mobile application adoption affect service access and daily integration, making digital literacy a key structural variable in cross-city older adult care (6). (39) examined the matching of older adult care supply and demand in the Guangdong-Hong Kong-Macao Greater Bay Area from a governance perspective. The study reveals that collaboration on health and older adult care services within the Bay Area still faces institutional barriers, including mutual recognition of qualifications, information sharing, and regulatory differences. This indicates that the matching of cross-city older adult(s) care supply and demand depends not only on the quantity of facilities but also on regional collaborative governance capabilities (39).

Guangzhou ranks among China’s megacities with the fastest aging pace and most pronounced spatial disparities, while also serving as a key older adult care destination within the Guangdong-Hong Kong-Macao Greater Bay Area. Data from the Seventh National Population Census indicate that the proportion of older adult residents reached 18.9 percent in Yuexiu District, one of the city’s oldest urban areas. In comparison, the corresponding figure in Nansha District was only 5.7 percent. This internal disparity exceeded that observed in Beijing and Shanghai during the same period (6). At the same time, Guangzhou has been designated as a national pilot city for reforms in home-based and community-based older adult care services. Since 2019, the municipal government has issued a series of policy documents, including community living circle plans and specialized plans for the spatial distribution of older adult care institutions. These initiatives have created an integrated research context combining policy support, data availability, and practical implementation scenarios (6, 31–34).

Within this context, recent studies have increasingly focused on the spatial equilibrium between older adult care service resources and demand in Guangzhou. Using multi-source data at the 500-meter grid level, Lai et al. (40) evaluated the spatial equity of public facility provision in the city. They found that old urban areas and urban villages exhibited clusters characterized by high social vulnerability and low comprehensive accessibility. These clusters showed substantial spatial overlap with areas of high older population concentration, providing a representative empirical setting characterized by high demand and insufficient resource provision. As such, Guangzhou offers a valuable case for examining the spatial equilibrium and disequilibrium of older adult care service resources and demand in large metropolitan contexts (32). Feng et al. (41) focused on the supply side of older adult care services in Guangzhou, employing POI and machine learning to optimize the layout of older adult care facilities. This research provides methodological support for spatially optimizing resource allocation and service coverage in high-density urban areas. The aforementioned study complements this paper by offering the latest evidence and comparative references for discussing supply–demand mismatches within the Guangzhou context.Beyond the Guangzhou context, a growing body of research has examined the spatial differentiation, accessibility, and health inequality dimensions of population aging across multiple Chinese regions (42–48). These studies collectively highlight pronounced spatial heterogeneity in aging distribution and service accessibility, reinforcing the necessity of integrating spatial equilibrium perspectives into analyses of care service supply–demand alignment

### 2.4 Literature review summary

Existing studies have examined the functioning of older adult care service systems from multiple perspectives, including spatial patterns, demand measurement, resource allocation, and the matching between service resources and demand. Research scales have gradually shifted toward community and living-circle levels, reflecting an increasing emphasis on microspatial analysis. However, within the context of megacities, several limitations remain evident. In cities with highly concentrated older populations and pronounced spatial heterogeneity, the equilibrium relationship between dynamically evolving older adult care service demand and existing resource allocation structures has not been sufficiently examined.

As a megacity experiencing rapid population aging and substantial internal disparities, Guangzhou is characterized by the combined effects of inner-city renewal, new district expansion, and sustained population mobility. Under these conditions, older adult care service demand exhibits marked spatial differentiation, while the extent to which current resource allocation structures can effectively respond to this differentiation remains unclear. Existing studies have not yet provided a systematic assessment of the equilibrium between older adult care service resources and demand within the city.

Accordingly, it is necessary to take Guangzhou as a case study and conduct a systematic examination of the equilibrium between older adult care service resources and demand at the community scale. Such an approach can provide empirical evidence to support more precise allocation of older adult care resources and contribute to the optimization of urban older adult care service systems.

## 3 Research methods and data description

### 3.1 Spatial autocorrelation analysis

Spatial autocorrelation refers to the degree of spatial dependence among observations across different geographic units, reflecting the extent to which attribute values are correlated with their spatial locations. In other words, it describes the dependence between observed values and spatial proximity, capturing patterns of mutual influence and interaction among neighboring areas. This approach has been widely applied in studies of population aging to examine spatial clustering and diffusion processes.

Moran’s *I* is a commonly used indicator for measuring spatial autocorrelation. It evaluates the similarity of attribute values among spatially adjacent or nearby decision units and is used to assess the degree of spatial clustering within a study area. A positive Moran’s *I* value indicates spatial clustering of similar values, whereas a negative value suggests spatial dispersion. The index is calculated as follows:


$$I=\frac{\sum_{i=1}^{n}\sum_{j=1}^{n}(x_{i}-\bar{x}\;)}{S^{2}\sum_{i=1}^{n}\sum_{j=1}^{n}w_{\mathrm{ij}}}\;$$ (1)

where $S^{2}$ denotes the sample variance, defined as $S^{2}=\frac{1}{n}\sum_{i=1}^{n}\;{\left(x_{i}-\bar{x}\right)}^{2}$ and $w_{\mathrm{ij}}\;\;$ represents the element of the spatial weight matrix corresponding to regions i and j , which is used to measure the spatial proximity between the two regions. The term $\sum_{i=1}^{n}\;\sum_{j=1}^{n}\;w_{\mathrm{ij}}$ denotes the sum of all spatial weights among the 11 administrative districts of Guangzhou.

The value of Moran’s *I* ranges from −1 to 1. A larger positive value indicates stronger positive spatial autocorrelation in population aging across regions, whereas a smaller or negative value suggests weaker spatial dependence or spatial dispersion.

Based on the calculated Moran’s *I*, statistical significance is assessed using a standardized normal distribution to test whether spatial autocorrelation exists among regions. The corresponding test statistic is calculated as follows:


$$Z(I)=\frac{{\text{Moran}}^{'}s-E(I)}{\sqrt{\mathrm{VAR}(I)}}$$ (2)

The presence of spatial autocorrelation in the development of population aging levels in Guangzhou is assessed based on the magnitude of the Z statistic. When the Z value is greater than or equal to zero and statistically significant, the development of population aging levels in Guangzhou exhibits positive spatial autocorrelation, indicating spatial homogeneity and positive spatial spillover effects. When the Z value is less than or equal to zero and statistically significant, negative spatial autocorrelation is observed, reflecting spatial heterogeneity across regions. When the Z value equals zero, the development of population aging levels across districts in Guangzhou follows a random spatial distribution.

### 3.2 Entropy weight method

The entropy weight method is used to assign indicator weights in an objective manner, thereby reducing the randomness associated with subjective weighting approaches. In studies related to older adult populations, evaluating and improving the efficiency and effectiveness of service systems is of particular importance. As an objective weighting technique, the entropy weight method has been widely applied in assessments of population aging development because it captures the information entropy of each indicator and determines weights based on the degree of variation in the data. Indicators with greater informational content are assigned higher weights, allowing the composite index to more accurately reflect differences in development levels.

### 3.3 Geographic concentration

To comprehensively account for population aging, older adult care service resources, and land area across regions, this study introduces the geographic concentration of older adult care service demand, denoted as $R_{\log_{i}}$ , and the geographic concentration of older adult care service resources, denoted as $R_{{\mathrm{res}}_{i}}$ . These indices are used to characterize the degree of spatial concentration of older adult care service demand and resources, respectively. By comparing their spatial distributions, the measures reveal the spatial matching relationship and structural characteristics between older adult care service demand and resources in Guangzhou. The corresponding calculation formulas are presented below.


$$R_{\log_{i}}=\frac{\frac{\log_{i}}{\sum \log_{i}}}{\frac{\mathrm{te}r_{i}}{\sum \mathrm{te}r_{i}}}\;\;$$ (3)

$$R_{{\mathrm{res}}_{i}}=\frac{\frac{{\mathrm{res}}_{i}}{\sum {\mathrm{res}}_{i}}}{\frac{{\mathrm{ter}}_{i}}{\sum {\mathrm{ter}}_{i}}}$$ (4)

In Equations 3 and 4, $\log_{i}$ represents the level of older adult care service demand in district i during a given period, and $\mathrm{te}r_{i}$ denotes the land area of district i . $\mathrm{re}s_{i}\;$ represents the level of older adult care service resources in district i . The terms $\sum \log_{i}$ , $\sum \mathrm{re}s_{i}$ , and $\sum \mathrm{te}r_{i}\;$ refer to the total older adult care service demand, total older adult care service resources, and total land area of Guangzhou, respectively.

The inconsistency index typically characterizes the relative deviation in the intensity of factor agglomeration between supply and demand by measuring the ratio of their concentration levels. Based on this, it forms a “leading-coordinated-lagging” typology to identify structural misallocations in public service provision (36, 37). Such indices have been widely used in international regional equilibrium studies to compare differences in factor agglomeration. This paper based on the geographic concentration indices, and drawing on related methodological approaches, this study uses the ratio of the geographic concentration of older adult care service demand $R_{\log_{i}}$ to the geographic concentration of older adult care service resources $R_{\mathrm{re}s_{i}}$ as an indicator to measure the degree of matching between older adult care service demand and resources. This indicator is denoted as RI . The corresponding calculation formula is given as follows:


$$\mathrm{RI}=\frac{R_{\log_{i}}}{R_{{\mathrm{res}}_{i}}}$$ (5)

In Equation 5, RI denotes the matching coefficient between older adult care service demand and older adult care service resources, also referred to as the inconsistency index. A smaller RI value indicates a stronger degree of resource concentration relative to demand. In general, when RI<1 , the concentration of older adult care service resources is ahead of the concentration of older adult care service demand. When RI=1 , the concentration of older adult care service resources is coordinated with the concentration of older adult care service demand. When RI>1 , the concentration of older adult care service resources lags behind the concentration of older adult care service demand.

Based on the inconsistency index, the degree of mismatch between older adult care service demand and resources can be classified as follows. When RI<1 , the concentration of older adult care service resources is ahead of that of older adult care service demand. When 1≤RI≤3 , the concentration levels of older adult care service resources and demand are considered coordinated. When RI>3 , the concentration of older adult care service resources significantly lags behind the concentration of older adult care service demand.

### 3.4 Evaluation indicator system and data sources

#### 3.4.1 Evaluation indicator system for the equilibrium of the older adult care system

The analysis of equilibrium in the older adult care system of Guangzhou focuses on the degree of matching between two subsystems, namely older adult care service demand and older adult care service resources. The level of equilibrium in resource allocation reflects the relationship between older adult care service demand and the level of resource input. Older adult care service resources comprise production factors such as capital and labor, and resource inputs include both hardware related elements and software related elements. Older adult care service demand represents a derived demand arising from the process of population aging and is influenced by factors such as the level of population aging and the spatial distribution density of the older population.

To characterize the level of population aging, this study employs two indicators: the older population coefficient and older population density (Table 1). The older population coefficient is operationally defined as the proportion (%) of permanent residents aged 60 years and above in each district, as reported in official population statistics. Older population density is defined as the number of residents aged 60 years and above per square kilometer of administrative land area (persons/km^2^), computed by dividing the district older population by the district land area (km^2^) published in official statistical sources. Because effective demand for older adult care services is also shaped by socioeconomic capacity, per capita disposable income is introduced to represent the level of socioeconomic development; it follows the official statistical caliber of per capita disposable income of permanent residents (yuan/person, current prices) released in the Guangzhou Statistical Yearbook and district statistical bulletins.

**Table 1:** Indicator system of older adult care service demand in Guangzhou.

Target level	Criterion level	Indicator level	Variable (unit)
older adult care service demand indicator system	Level of population aging development	older population coefficient	% (X1)
		older population density	persons per square kilometer (X2)
	Level of socioeconomic development	Per capita disposable income	yuan

older adult care resources are measured across four dimensions: facility provision, community and home-based support, integrated service hubs, and medical support capacity. Facility resources include: the number of older adult care institutions per thousand people and the number of beds in older adult care institutions per thousand people. Community and home-based service resources characterize home-community care support capacity, including: the number of community meal assistance facilities per thousand seniors, legally registered or officially announced “senior dining halls/meal assistance points,” and the number of institutions providing community-based home care bed services per thousand seniors, that is, “home care bed” service providers registered or announced by civil affairs departments. Comprehensive service resources indicate the capacity of integrated older adult care service hubs, measured by the number of district-level comprehensive older adult care service centers and subdistrict/township-level comprehensive older adult care service centers, both uniformly converted to per thousand indicators. Medical service resources characterize the medical support capacity for older adult care services, including: the number of medical institutions, the number of medical institution beds, and the number of healthcare professionals, all calculated per thousand people. The denominator for all “per thousand” indicators uses the district-level resident population for the corresponding year. The complete indicator system is shown in Table 2.

**Table 2:** Evaluation indicator system of older adult care service resources in Guangzhou.

Target level	Criterion level	Indicator level	Variable (unit)
older adult care service resource indicator system	A1 infrastructure resources	Number of older adult care institutions per 1,000 persons	per 1,000 persons (X3)
		Number of older adult care beds per 1,000 persons	beds per 1,000 persons (X4)
	A2 community and family-based service resources	Number of community meal service facilities for older adults per 1,000 persons	per 1,000 persons (X5)
		Number of service institutions providing community-based family care beds	per 1,000 persons (X6)
	A3 comprehensive service system resources	Number of district level comprehensive older adult care service centers per 1,000 persons	per 1,000 persons (X7)
		Number of subdistrict or township level comprehensive older adult care service centers per 1,000 persons	per 1,000 persons (X8)
	A4 medical service system resources	Number of medical institutions per 1,000 persons	per 1,000 persons (X9)
		Number of healthcare beds per 1,000 persons	beds per 1,000 persons
		Number of health technical personnel per 1,000 persons	persons per 1,000 persons

Data were compiled and processed by the authors.

#### 3.4.2 Data sources

The data used in this study primarily originates from the Guangzhou Statistical Yearbook and statistical bulletins released by various districts. Supplementary facility data, the number of older adult care institutions, bed capacity, community meal service facilities, home care bed service providers, and integrated service centers was compiled from publicly released announcements by the Guangzhou Civil Affairs Bureau and district civil affairs departments.

Data collection and processing followed a transparent workflow: (1) district-level population and socioeconomic variables older population, permanent residents, per capita disposable income, land area were extracted from the 2015, 2019, and 2023 Guangzhou Statistical Yearbooks and district statistical bulletins; (2) older adult care institutions and service resource variables were sourced from corresponding-year civil affairs public data, annual reports, and official announcements; (3) all entries underwent multi-source cross-verification; discrepancies were resolved by adopting the most authoritative official data for that year with corresponding annotations; (4) raw counts were converted to per-thousand indicators based on each district’s permanent resident population, and density metrics were calculated using officially designated land area; (5) The final dataset was organized in a “district-year” panel format for entropy weighting, composite index construction, and subsequent mismatch analysis.

## 4 Empirical results

### 4.1 Spatial differences in population aging across districts in Guangzhou

Based on the calculation of the older population coefficient and older population density for the 11 districts of Guangzhou in 2015, 2019, and 2023, this section examines the spatial differentiation of population aging across the city. With reference to international and domestic standards on population aging as well as related empirical studies, population aging levels are classified into five categories: areas with an older population proportion below 5 percent are defined as young type; 5 to below 7 percent as adult type; 7 to below 10 percent as early aging type; 10 to below 14 percent as moderate aging type; and 14 percent and above as deep aging type. By integrating these classifications with the spatial distribution of population aging, differences in aging stages and temporal changes across districts are compared.

**Figure 1:**
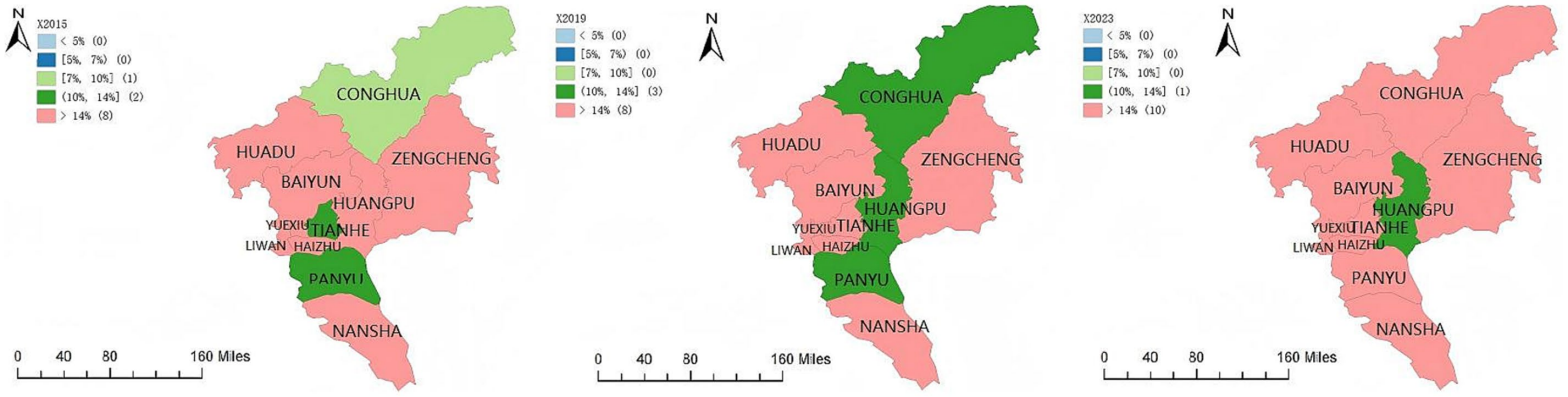
Trends in population aging in Guangzhou. Maps created using ArcGIS (Esri).

As shown in Figure 1 and Table 3, Guangzhou had already entered a relatively advanced stage of population aging in 2015. No districts fell into the young or adult categories. Conghua was classified as early aging and represented the youngest district in terms of age structure. Tianhe and Panyu had entered the stage of moderate aging, while the remaining eight districts, including Huadu, Zengcheng, Baiyun, Huangpu, Yuexiu, Haizhu, Liwan, and Nansha, were classified as deep aging areas.

**Table 3:** older population coefficient and older population density across districts of Guangzhou.

District	Year older population coefficient (%) older population density (persons per square kilometer)					
	2015	2019	2023	2015	2019	2023
Liwan	24.49	28.46	31.13	2,988	3,640	4,239
Yuexiu	22.57	26.18	29.39	7,845	9,085	10,175
Haizhu	22.25	25.75	28.72	2,487	3,039	3,526
Tianhe	13.01	14.19	15.39	1,141	1,423	1747
Baiyun	16.41	16.7	17.23	189	227	266
Huangpu	14.03	13.19	12.93	127	153	188
Panyu	13.55	13.71	14.31	219	267	325
Huadu	15.22	15.32	15.45	111	128	144
Nansha	16.43	16.14	15.95	80	95	115
Conghua	12.42	13.5	15.06	39	44	51
Zengchen	14.16	14.15	14.44	76	86	100

Data were obtained from the Guangzhou Statistical Yearbook and district-level statistical bulletins, with supplementary information from older adult care resource data released by the Guangzhou Civil Affairs Bureau in 2023. The table was compiled and processed by the authors.

By 2019, changes in the degree of population aging were observed. Huangpu transitioned from deep aging to moderate aging, and Conghua moved from early aging to moderate aging. Panyu, Conghua, and Huangpu were classified as moderate aging districts, whereas Huadu, Zengcheng, Baiyun, Yuexiu, Haizhu, Liwan, Nansha, and Tianhe remained in the deep aging category. At the citywide level, no districts were classified as young, adult, or early aging.

In 2023, population aging in Guangzhou further intensified. Panyu and Conghua transitioned from moderate aging to deep aging. Apart from Huangpu, which remained at the moderate aging stage, all other districts were classified as deep aging areas.

Using older population density as an indicator, this section compares the degree of spatial concentration and regional differences in the distribution of the older population. Older population density is classified into five levels: less than 20 persons per square kilometer indicates low density; 20 to less than 40 persons per square kilometer indicates relatively low density; 40 to less than 80 persons per square kilometer indicates medium density; 80 to 100 persons per square kilometer indicates relatively high density; and greater than 100 persons per square kilometer indicates high density.

**Figure 2:**
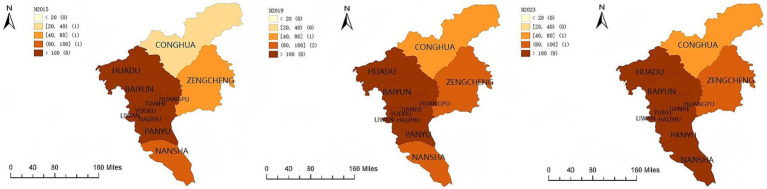
Spatial distribution of older population density in Guangzhou. Maps created using ArcGIS (Esri).

As shown in Figure 2, older population density in Guangzhou exhibited a slight overall increase from 2015 to 2023. In 2015, Conghua District recorded an older population density of 39 persons per square kilometer and was classified as a relatively low-density area. Zengcheng District, with 76 persons per square kilometer, and Nansha District, with 80 persons per square kilometer, were classified as medium density areas. The remaining eight districts were classified as high density areas, each with more than 100 older persons per square kilometer.

By 2019, older population density increased further across the city. Conghua transitioned from a relatively low-density area to a medium density area, while Zengcheng and Nansha shifted from medium density to relatively high-density categories. The classification of the remaining eight districts remained unchanged. In 2023, Nansha transitioned from a relatively high-density area to a high-density area, while no substantial changes were observed in the other districts. Overall, the city comprised nine high density districts, one medium density district, and one relatively high-density district.

### 4.2 Spatial association characteristics of population aging in Guangzhou

**Figure 3:**
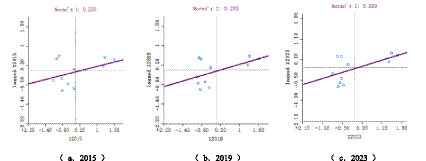
LISA cluster map of population aging across districts in Guangzhou.

To further elucidate regional differences in the degree of population aging in Guangzhou, exploratory spatial data analysis (ESDA) was conducted using ArcGIS, with districts serving as the basic spatial units. From a spatial perspective According to Equations (1) and (2), this analysis examines the patterns of spatial differentiation and clustering in population aging across districts. Global Moran’s *I* statistics of the older population coefficient were calculated for 2015, 2019, and 2023 to assess the evolution of spatial dependence in population aging. The results are presented in Table 4 and Figure 3.

**Table 4:** Global Moran’s *I* Index and related statistics of population aging in Guangzhou.

Year	Moran’s *I*	*z*-value	*p*-value
2015	0.230	1.8571	0.045
2019	0.283	2.0289	0.039
2023	0.299	2.1518	0.035

The Global Moran’s *I* values were positive for all 3 years and increased slightly over time. All estimates passed the statistical significance tests, indicating the presence of positive spatial autocorrelation in population aging across districts in Guangzhou. Higher Moran’s *I* values closer to 1 reflect stronger positive spatial dependence. These results suggest that districts with similar levels of population aging tend to be spatially clustered, with high aging areas adjacent to other high aging areas and lower aging areas exhibiting similar spatial proximity. Overall, population aging in Guangzhou displays a clear pattern of spatial clustering, and this clustering effect has strengthened over time.

Comparative analysis indicates that these spatial association patterns are closely related to changes in the natural population growth rate, the growth rate of the older adult population, and population migration across districts. In summary, from 2015 to 2023, population aging in Guangzhou exhibited a significant positive spatial association at the district level, while disparities in aging development across districts showed a tendency to widen over time.

**Figure 4:**
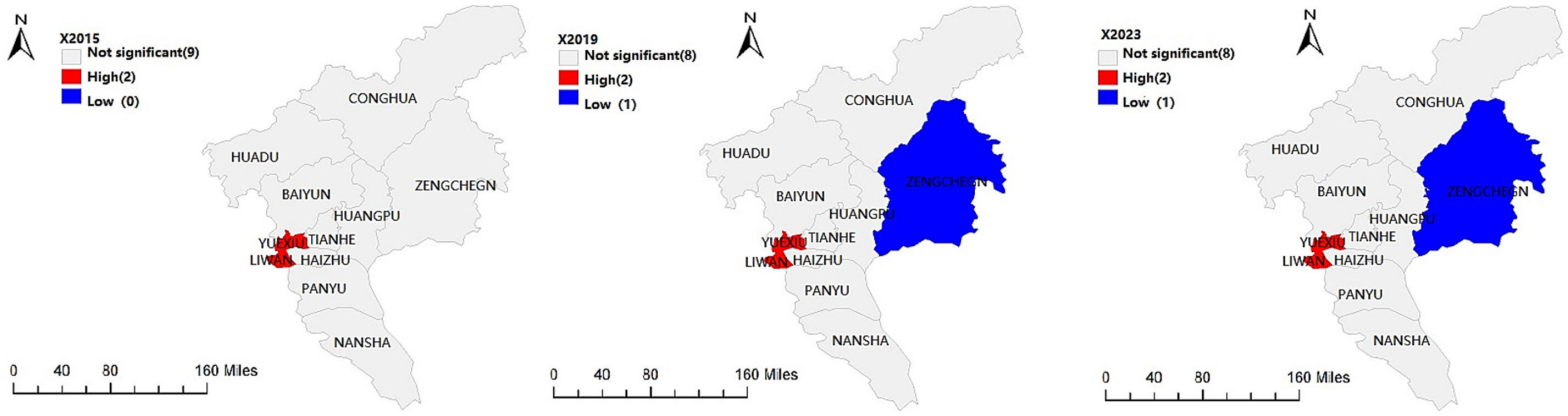
Hot and cold spot distribution of population aging across districts of Guangzhou in 2015, 2019, and 2023. Maps created using ArcGIS (Esri).

To further investigate the specific spatial distribution of population aging clusters in Guangzhou, local spatial autocorrelation analysis was conducted using GeoDa. This analysis examines the spatial association and heterogeneity of population aging levels between each district and its neighboring districts and reveals the spatial patterns and their temporal characteristics. The results are illustrated in Figure 4.

In 2015, the high value clusters of population aging in Guangzhou were concentrated in Yuexiu and Liwan Districts, forming distinct hot spots, while no low value clusters were identified. Compared with 2015, a new low value cluster emerged in Zengcheng District in eastern Guangzhou in 2019, whereas the high value clusters remained unchanged in Yuexiu and Liwan Districts. The spatial pattern observed in 2023 was consistent with that in 2019. Zengcheng continued to exhibit a relatively low level of population aging and maintained a comparatively younger population structure.

Overall, the spatial pattern of population aging in Guangzhou is characterized by stable hot spots persistently located in Yuexiu and Liwan Districts, while cold spots are primarily distributed in the eastern part of the city. Multiple factors contribute to the spatial distribution and temporal stability of hot and cold spots, among which regional economic development level and population migration play dominant roles. Areas with higher levels of economic development tend to exhibit more pronounced population aging. The central urban districts of Guangzhou, characterized by advanced economic development, well developed infrastructure, and favorable medical conditions, constitute important destinations for older residents. In contrast, although eastern Guangzhou possesses relatively favorable natural environments, comparatively lower levels of economic development, medical services, and infrastructure may limit its attractiveness for older adult care settlement.

## 5 Regional differences in the allocation of older adult care service resources in Guangzhou

### 5.1 Spatial matching between older adult care service demand and resource allocation in Guangzhou

#### 5.1.1 Regional differences in older adult care service resource allocation across districts

Based on Equations 3–5, the entropy weight method was applied to calculate the weights of the older adult care service demand indicators. A comprehensive evaluation model was then constructed to estimate the composite scores and rankings of older adult care service demand for the resident population across districts in Guangzhou. The results are presented in Table 5.

**Table 5:** Composite scores and ranking of older adult care service demand across districts of Guangzhou in 2020.

District	2015	2019	2023	Mean	Rank
Liwan	0.5914	0.6444	0.6254	0.6204	2
Yuexiu	0.9548	0.9392	0.9603	0.9514	1
Haizhu	0.4936	0.5413	0.5458	0.5269	3
Tianhe	0.2063	0.1982	0.2392	0.2146	4
Baiyun	0.1771	0.1574	0.1534	0.1626	5
Huangpu	0.1192	0.0723	0.0874	0.0930	7
Panyu	0.1038	0.0792	0.0951	0.0927	8
Huadu	0.1092	0.0912	0.0829	0.0944	6
Nansha	0.1249	0.1021	0.0885	0.1052	9
Conghua	0.0001	0.0072	0.0363	0.0145	11
Zengcheng	0.0653	0.0462	0.0555	0.0557	10

Data were obtained from the Guangzhou Statistical Yearbook and older adult care resource data released by the Civil Affairs Department in 2023. Figures and tables were compiled and prepared by the authors.

The results indicate substantial regional disparities in older adult care service demand across districts in Guangzhou. Central districts characterized by higher levels of economic development and more advanced population aging exhibited the highest overall demand. The top three districts were Yuexiu, Liwan, and Haizhu, all located in the central urban area, with average demand scores of 0.9514, 0.6204, and 0.5269, respectively.

In contrast, districts with lower levels of economic development and younger population structures showed relatively low demand. Nansha District in southern Guangzhou ranked ninth, with an average demand score of 0.1054. Zengcheng District in eastern Guangzhou ranked tenth, with a score of 0.0557. Conghua District exhibited the lowest level of older adult care service demand, ranking twelfth, with an average score of 0.0145. Overall, the level of older adult care service demand reflects, to a certain extent, interdistrict differences in economic development and the degree of population aging.

#### 5.1.2 Regional differences in older adult care service resource allocation across districts

Using the entropy weight method, weights were calculated for each older adult care service resource indicator. A comprehensive evaluation model was then constructed to estimate the composite scores and rankings of older adult care service resource allocation for the resident population across districts in Guangzhou. The results are presented in Table 6.

**Table 6:** Composite scores and ranking of older adult care service resource allocation across districts of Guangzhou in 2023.

District	Infrastructure resources	Community service resources	Comprehensive service system resources	Medical service system resources	Composite score	Rank
Liwan	0.5391	0.1438	0.1977	0.0327	0.2500	8
Yuexiu	0.0222	0.1434	0.0001	0.4784	0.1777	10
Haizhu	0.1766	0.0604	0.0294	0.1469	0.0963	11
Tianhe	0.1985	0.3960	0.3794	0.8698	0.5576	3
Baiyun	0.8707	0.3785	0.7194	0.8243	0.8481	1
Huangpu	0.8311	0.7822	0.6739	0.4830	0.8446	2
Panyu	0.3533	0.3451	0.4088	0.3011	0.4075	5
Huadu	0.3431	0.1394	0.5737	0.2856	0.3756	6
Nansha	0.2925	0.4949	0.6527	0.1129	0.4437	4
Conghua	0.2630	0.5233	0.3039	0.1286	0.3514	7
Zengcheng	0.0517	0.2784	0.3057	0.2722	0.2492	9

Data were obtained from the Guangzhou Statistical Yearbook and older adult care resource data released by the Civil Affairs Department in 2023.

Source: authors.

The results reveal substantial regional disparities in the level of older adult care service resource allocation across districts in Guangzhou. Baiyun District and Huangpu District exhibited the highest overall levels of resource allocation, indicating relatively abundant older adult care service resources. In contrast, Yuexiu District and Haizhu District showed the lowest levels of older adult care service resource allocation.

To a certain extent, the level of older adult care service resource allocation reflects interdistrict differences in fiscal investment and the intensity of policy support.

### 5.2 Geographic concentration of older adult care service demand and resources in Guangzhou

#### 5.2.1 Geographic concentration of older adult care service demand and resources

Using data on older adult care service demand and older adult care service resources for 2023, this study calculates the geographic concentration indices of demand and resources, as well as the inconsistency index, based on Equations 3–5. The results are reported in Table 7.

**Table 7:** Geographic concentration of older adult care service demand and resources and the inconsistency index in Guangzhou.

District	Older adult care service demand	Older adult care service resources	Area (km^2^)	Demand concentration index	Resource concentration index	Inconsistency index (RI)
Liwan	0.6254	0.2500	59.1000	26.4904	0.0021	12916.8474
Yuexiu	0.9603	0.1777	33.8000	71.1228	0.0005	130993.2068
Haizhu	0.5458	0.0963	90.4000	15.1142	0.0014	10915.9028
Tianhe	0.2392	0.5576	96.3300	6.2161	0.0195	318.8836
Baiyun	0.1534	0.8481	795.7900	0.4826	0.3819	1.2635
Huangpu	0.0874	0.8446	484.1700	0.4519	0.4062	1.1126
Panyu	0.0951	0.4075	529.9400	0.4492	0.1971	2.2790
Huadu	0.0829	0.3756	970.0400	0.2139	0.3815	0.5607
Nansha	0.0885	0.4437	783.8600	0.2826	0.3412	0.8285
Conghua	0.0363	0.3514	1974.5000	0.0460	1.6593	0.0277
Zengcheng	0.0555	0.2492	1616.4700	0.0859	0.6301	0.1364

Data were obtained from the Guangzhou Statistical Yearbook and older adult care resource data released by the Guangzhou Municipal Civil Affairs Bureau in 2023.

Source: authors.

#### 5.2.2 Geographic concentration of older adult care service demand in Guangzhou

**Figure 5:**
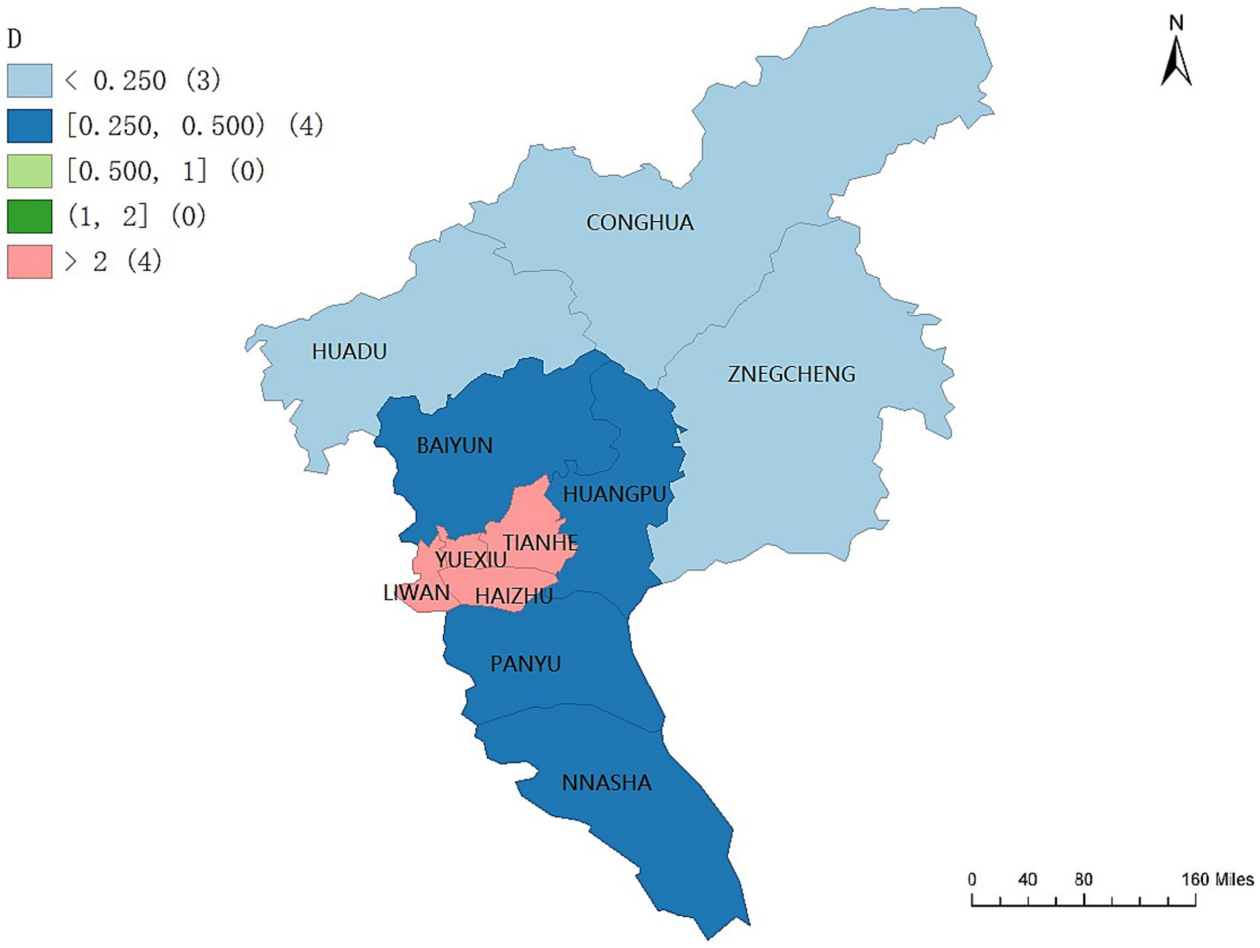
Geographic concentration of older adult care service demand across the 11 districts of Guangzhou. Maps created using ArcGIS (Esri).

The geographic concentration of older adult care service demand in Guangzhou exhibits an overall pattern characterized by high concentration in central districts and lower concentration in peripheral areas, as illustrated in Figure 5. Districts with highly concentrated older adult care service demand are primarily located in the central urban area, including Yuexiu, Liwan, Haizhu, and Tianhe. The demand concentration indices in these districts exceed 2, with values of 71.1228, 26.4904, 15.1142, and 6.2161, respectively.

Districts surrounding the core area, including Baiyun, Panyu, Nansha, and Huangpu, display moderate levels of demand concentration, with concentration indices ranging between 0.25 and 0.50. In contrast, districts located on the urban periphery, namely Zengcheng, Conghua, and Huadu, exhibit relatively dispersed patterns of older adult care service demand, with concentration indices below 0.25.

#### 5.2.3 Geographic concentration of older adult care service resources in Guangzhou

**Figure 6:**
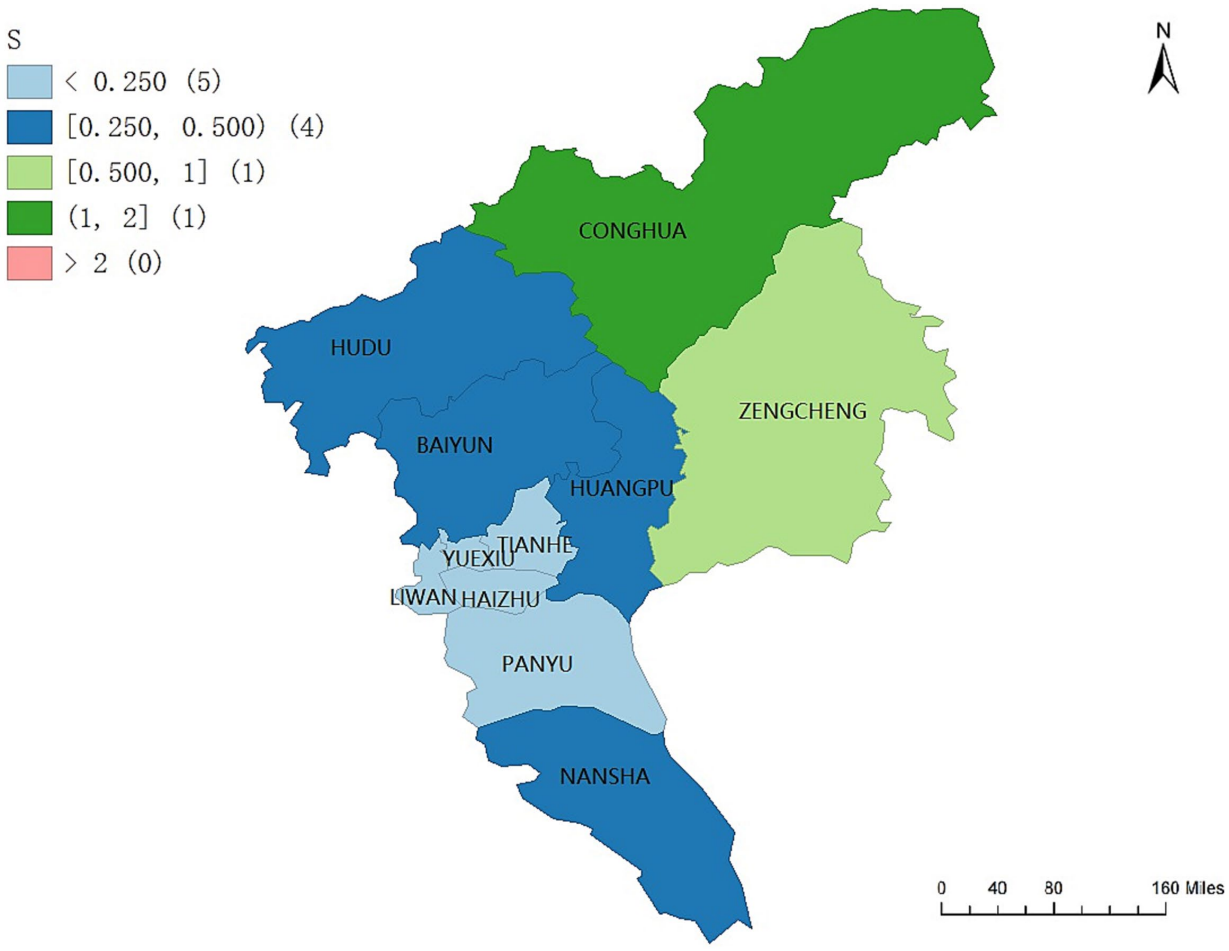
Geographic concentration of older adult care service resources across the 11 districts of Guangzhou. Maps created using ArcGIS (Esri).

The overall spatial pattern of the geographic concentration of older adult care service resources in Guangzhou exhibits a differentiated and tiered distribution, characterized by lower concentration in central districts, higher concentration in transitional districts, and the highest concentration in peripheral districts. As shown in Figure 6, the highest levels of resource concentration are observed in the peripheral districts of Conghua and Zengcheng, with concentration indices of 1.6593 and 0.6301, respectively.

In contrast, central districts with the highest levels of older adult care service demand, including Yuexiu, Liwan, Haizhu, Tianhe, and Panyu, display relatively low levels of resource concentration. The resource concentration indices of these five districts are all below 0.250. Districts located in the intermediate zone, namely Huadu, Baiyun, Huangpu, and Nansha, exhibit moderate levels of resource concentration, with indices ranging between 0.5 and 1.0.

#### 5.2.4 Correlation between the geographic concentration of older adult care service demand and resources

**Figure 7:**
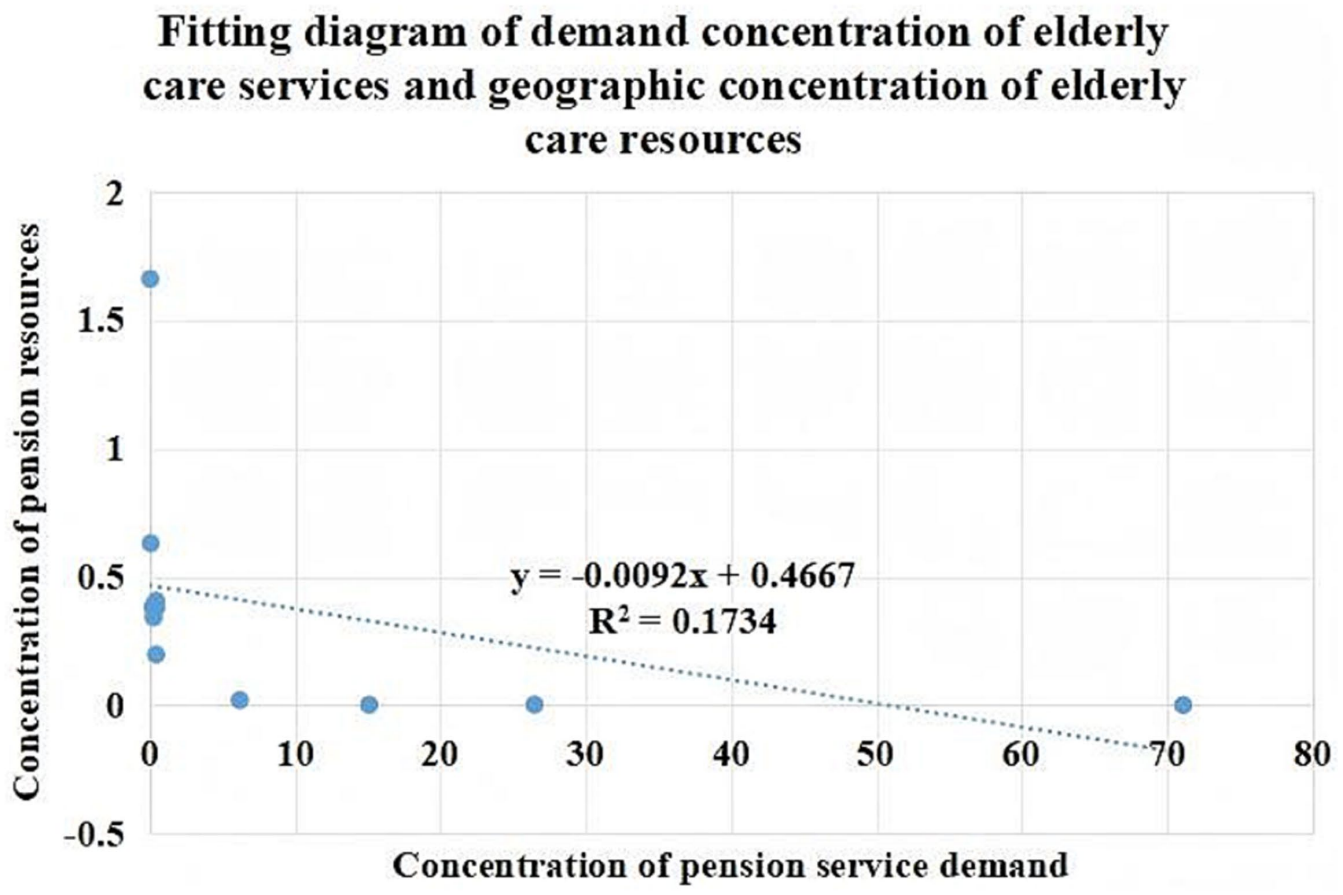
Analysis of geographic concentration of older adult care service demand and older adult care resources in Guangzhou. Maps created using ArcGIS (Esri).

To examine the relationship between the geographic concentration of older adult care service demand and older adult care service resources across districts in Guangzhou, a scatter plot was constructed, and a fitted curve was applied, as shown in Figure 7. The scatter plot clearly illustrates the distribution patterns of demand and resources and reveals a low to moderate negative correlation between the two.

As reported in Table 8, the Pearson correlation coefficient between the geographic concentration of older adult care service demand and resources is −0.4146, indicating a negative association. The coefficient of determination (*R*^2^) is 0.1734, suggesting a relatively low degree of fit between the scatter points and the fitted curve. This result further confirms the presence of a negative relationship between older adult care service demand and resource concentration.

**Table 8:** Correlation analysis between the geographic concentration of older adult care service demand and resources in Guangzhou.

Correlation		Geographic concentration of older adult care service demand	Geographic concentration of older adult care service resources
Geographic concentration of older adult care service demand	Pearson correlation	1	−0.4146
	Sig. (two-tailed)	0.000	0.203
	N	11	11
Geographic concentration of older adult care service resources	Pearson correlation	−0.4146	1
	Sig. (two-tailed)	0.203	0.000
	N	11	11

Correlation is not significant at the 0.01 level (two-tailed).

The observed low negative correlation indicates the existence of spatial mismatch between older adult care service demand and resource allocation in Guangzhou, whereby areas with higher demand do not necessarily correspond to areas with higher levels of resource concentration.

### 5.3 Matching types of older adult care service demand and resources in Guangzhou based on the inconsistency index

Given that the correlation between the geographic concentration of older adult care service demand and that of older adult care service resources in Guangzhou is not pronounced, the inconsistency index (RI) is introduced to further clarify the matching relationship between demand and resources across the 11 districts. The RI index is used to assess the degree of matching between older adult care service demand and resource allocation and to identify spatial mismatch patterns.

**Figure 8:**
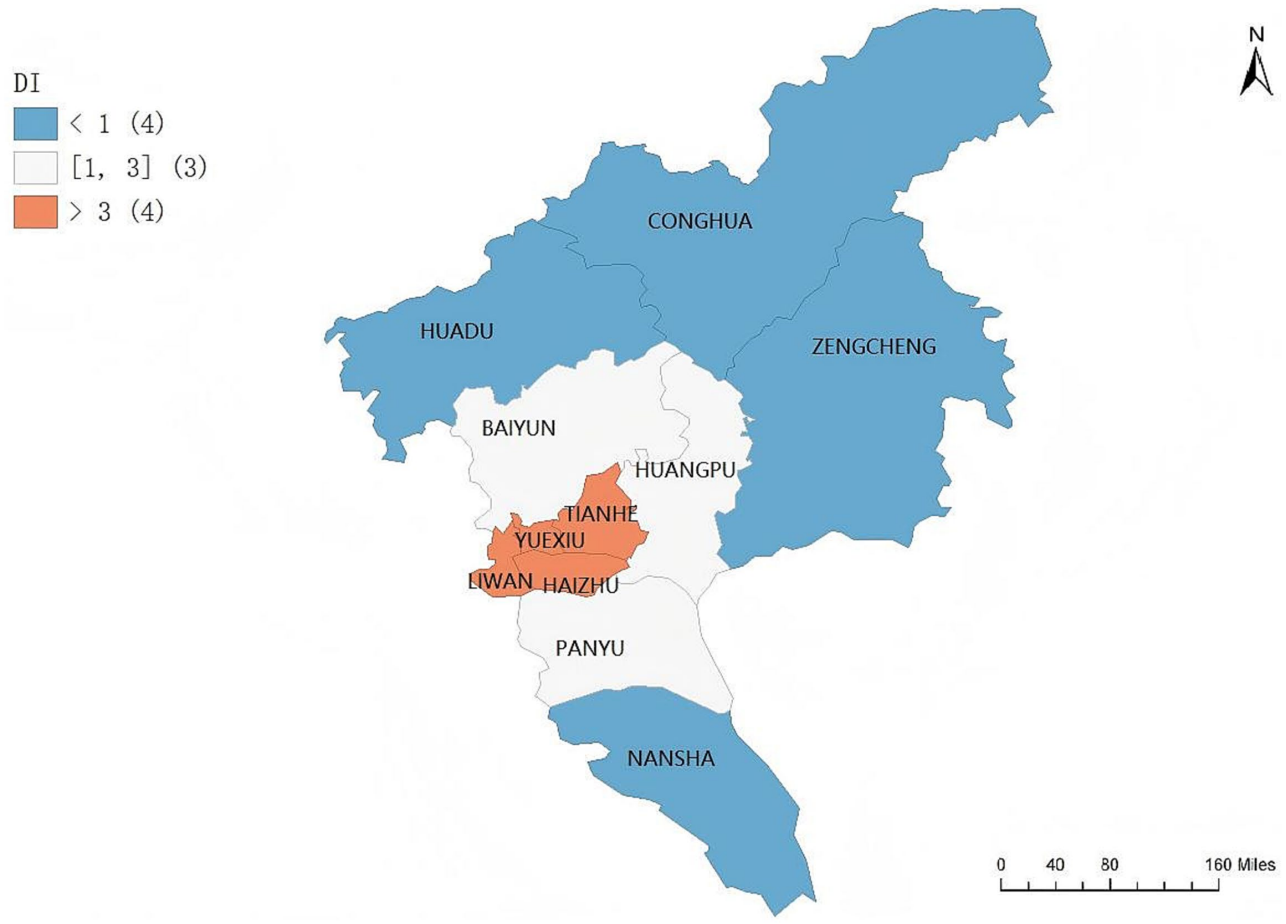
Distribution of the inconsistency index between older adult care service demand and resources across the 11 districts of Guangzhou.

Based on the inconsistency index, the equilibrium between older adult care service demand and resources in Guangzhou is classified into three matching types: (1) resource concentration ahead of demand concentration, (2) coordinated concentration of resources and demand, and (3) resource concentration lagging behind demand concentration. Specifically, when RI < 1, the concentration of older adult care service resources is ahead of the concentration of older adult care service demand. When 1 ≤ RI ≤ 3, the concentration levels of older adult care service resources and demand are considered coordinated. When RI > 3, the concentration of older adult care service resources lags behind the concentration of older adult care service demand. The spatial distribution of these matching types across districts is illustrated in the corresponding Figure 8.

#### 5.3.1 High-demand–low-supply “bottleneck areas” of older adult care services

The central districts of Guangzhou are subject to a dual constraint characterized by excessive demand and insufficient resource supply. Yuexiu, Liwan, Haizhu, and Tianhe constitute the core areas with highly concentrated older adult care service demand in Guangzhou. The demand scores for these four districts are 0.9603, 0.6254, 0.5458, and 6.2161, respectively, whereas their corresponding resource scores are only 0.1777, 0.2500, 0.0963, and 0.0195. As a result, the inconsistency index values reach 130,993.2068, 12,916.8474, 10,915.9028, and 318.8826, respectively, far exceeding the citywide average.

These findings indicate a pronounced decoupling between the spatial allocation of older adult care service resources and the growth trajectory of population aging in the central urban districts, giving rise to a structural contradiction characterized by extremely concentrated demand and severe resource scarcity.

The mechanisms underlying this imbalance can be summarized as follows. First, older adult residents in central districts exhibit strong residential inertia, with pronounced intergenerational co-residence patterns and relatively low willingness to relocate. Second, high land development intensity and limited community space impose dual constraints on the expansion of older adult care facilities, including land availability and planning approval. Third, fiscal investment is more heavily directed toward the maintenance of existing infrastructure rather than capacity expansion, resulting in resource provision that fails to keep pace with rising demand. Consequently, the interaction between older adult population concentration and spatial constraints on resource allocation leads to the formation of typical high-demand–low-supply bottleneck areas in the provision of older adult care services.

#### 5.3.2 Areas with resource-led allocation and lagging demand

In peripheral districts, the supply–demand relationship exhibits another extreme pattern, in which older adult care service resources significantly precede actual demand, forming a spatial configuration characterized by high resource levels and low demand. Peripheral areas such as Conghua District, Zengcheng District, Huangpu District, Huadu District, and Nansha District generally display resource concentration levels that are higher than demand concentration levels, with inconsistency index values significantly below 1, and in some areas even below 0.02. The inconsistency index of Conghua District is only 0.0277, and that of Zengcheng District is 0.1364, indicating that the supply of older adult care service resources in these areas significantly precedes the growth of demand.

Taking Conghua District as an example, the resource concentration index reaches 1.6593, while the demand concentration index is only 0.0460, showing a typical “resource spillover–type” characteristic. This pattern can be attributed to Guangzhou’s implementation of an “outward expansion–oriented resource allocation strategy” during the 13th and 14th Five-Year Plan periods, whereby large-scale comprehensive older adult care complexes were constructed in peripheral areas through land expansion in order to alleviate supply–demand pressure under the constraints of central urban areas. Although this strategy increased the total amount of resources in peripheral districts, the spatial center of the older adult population has not undergone a substantial shift, resulting in insufficient resource utilization efficiency and the emergence of facility vacancy in some areas.

The spatial lag effect is most pronounced in Zengcheng and Conghua. In these two districts, the resource concentration indices are 0.6301 and 1.6593, respectively, while the demand concentration indices are only 0.0859 and 0.0460, forming an evident spatiotemporal disjunction characterized by “resource precedence and demand lag.”

### 5.4 Spatial transitional zone with coordinated allocation

In the intermediate belt between the central and peripheral areas, Baiyun District, Panyu District, and Huangpu District exhibit relatively coordinated supply–demand conditions. Their inconsistency index values are 1.2635, 2.2790, and 1.1126, respectively, indicating that although differences between resources and demand exist, no extreme imbalance has occurred. Baiyun District and Huangpu District show relatively coordinated matching relationships between supply and demand. Their inconsistency index values are 1.2635 and 1.1126, respectively, and the gaps between resource concentration and demand concentration are relatively small, reflecting a relatively balanced supply state.

This relative balance benefits from the transitional location of these districts between the central urban area and the peripheral area. They possess both industrial carrying capacity and population attractiveness, featuring a certain foundation of population aging while also having strong potential for resource expansion. Baiyun District records an older adult care resource score as high as 0.8481, the highest value in the city, indicating that it has formed a relatively complete system in terms of facility construction, community services, and medical support. Meanwhile, its demand concentration index of 0.4826 suggests that the spatial distribution of the older adult population is relatively dispersed, with no severe local saturation. Huangpu District shows a similar pattern. It has made significant investments in integrated medical and older adult care resources, forming a health and older adult care industry chain linked with the development of science and technology parks, and thus demonstrates a relatively high level of coordination.

From a citywide perspective, the distribution of the inconsistency index exhibits a strong bipolar pattern. At one end are districts represented by Yuexiu, Liwan, and Haizhu, which are characterized as “high-demand–low-resource” areas, with inconsistency index values reaching several thousand or even tens of thousands, indicating extremely high service pressure. At the other end are districts represented by Conghua and Zengcheng, which are characterized as “high-resource–low-demand” areas, with inconsistency index values below 0.2, indicating that resource investment significantly precedes demand.

This polarization not only reflects an imbalance in spatial structure but also reveals the social consequences of urban functional distribution. The spatial separation between the residential locations of the older population and the layout of older adult care facilities makes it difficult for the social care system to form effective spatial linkage, thereby reducing the overall efficiency of older adult care services in the city.

In summary, the spatial structure of older adult care resource allocation in Guangzhou has entered a stage of structural transformation. Its characteristics have shifted from the previous pattern of “overconcentration in the center and scarcity in the periphery” to a pattern of “saturation in the center and advancement in the periphery,” while the geographic mismatch between supply and demand has not yet been fundamentally resolved. High inconsistency index values reflect the service crisis of an aging society in central areas, whereas low index values reveal concerns about resource accumulation and potential waste. In the future, the key to older adult care policy in Guangzhou does not lie in simply increasing the total amount of resources, but in achieving dynamic spatial balance, functional complementarity in structure, and cross-district coordination at the institutional level, so as to build a high-quality older adult care service system that combines equity and efficiency.

## 6 Conclusions and recommendations

### 6.1 Conclusion

Based on a comprehensive analysis of the spatial distribution of population aging and older adult care resources in Guangzhou from 2015 to 2023, this study draws the following empirical conclusions. The level of population aging in Guangzhou has shown a continuously deepening trend, and its spatial pattern exhibits significant positive clustering characteristics. The central urban districts are the areas with the most pronounced population aging in the city. Peripheral areas have relatively younger population structures; however, the trend of aging diffusion is evident, and the spatial clustering effect of population aging has been continuously strengthening.

A typical spatial mismatch exists between older adult care service demand and resource allocation. The citywide older adult care service system presents a structural gradient characterized by “overloaded demand in the center, redundant supply in the periphery, and coordinated buffering in intermediate areas.” The central urban districts constitute urban “older adult care bottleneck areas,” while peripheral districts exhibit a “resource-leading” pattern, accompanied by issues such as low service utilization rates, resource accumulation, and spatial redundancy.

### 6.2 Development recommendations

The development of the older adult care system in Guangzhou is shifting from quantity-oriented expansion toward quality-oriented coordination. The key focus in the future does not lie in simply increasing the total number of facilities, but in achieving spatial balance of resources, precise service provision, and intelligent upgrading of the governance system. High-quality development of the older adult care system should be promoted from three dimensions: spatial optimization, structural integration, and institutional innovation.

First, the “micro-space supply enhancement” approach in central urban districts should be strengthened. Through the construction of embedded community-based older care centers, subdistrict-level service stations, and integrated medical–older adult care service points, land use efficiency and facility density can be improved, thereby alleviating supply shortages in high-demand areas. Second, peripheral districts should be guided to shift from resource expansion toward functional linkage transformation. Cross-district resource-sharing mechanisms should be established, and connectivity among transportation, medical services, and digital platforms should be enhanced, enabling peripheral resources to effectively accommodate spillover demand from central areas. Third, a citywide dynamic monitoring and spatial decision-making system for older adult care resources should be improved. By using GIS and big data technologies to construct “supply–demand heat maps,” resource allocation can be made more precise and forward-looking. Fourth, social capital and market entities should be encouraged to participate, forming a diversified older adult care framework characterized by government guidance, enterprise collaboration, and community co-governance.

Through the construction of a multi-level, wide-coverage, and digitalized older adult care service network, coordinated advancement can be achieved between population structure transformation and the restructuring of the urban service system.

### 6.3 Research limitations and future directions

This study examines the spatial mismatch between older adult care demand and resource allocation in Guangzhou from 2015 to 2023 at the district administrative level. It employs a combined framework of entropy weighting, spatial autocorrelation, and geographic concentration/dispersion indices to identify structural patterns in Guangzhou’s aging trends and resource allocation. Key limitations include: Indicators relied on statistical yearbooks and publicly available civil affairs data to construct supply–demand panels, excluding behavioral variables such as actual facility utilization rates, health status, and migration intentions from mechanism discussions. Methodologically, resource and demand concentration was measured based on area while incorporating traffic impedance and service radius. Future research could integrate POI and road network data at the subdistrict/grid unit level, employing accessibility models like E2SFCA and spatiotemporal heterogeneity methods to characterize “accessible supply of older adult care resources”. Concurrently, incorporating survey and platform operational data could validate migration intentions and service utilization mechanisms. Combining policy texts with process-tracking evaluation tools to assess implementation biases would establish a progressive research framework: “mismatch identification—mechanism validation—policy evaluation.”

## Data Availability

The original contributions presented in the study are included in the article/supplementary material, further inquiries can be directed to the corresponding author.

## References

[1] Wang M Shaobin W Hao Y. Exploring the spatial-temporal distribution and evolution of population aging and social-economic indicators in China. BMC Public Health. (2021) 21:966. doi: 10.1186/S12889-021-11032-Z 34020620 PMC8140474

[2] Xu X Zhao Y Zhang X Xia S. Identifying the impacts of social, economic, and environmental factors on population aging in the Yangtze River Delta using the geographical detector technique. Sustainability. (2018) 10:1528. doi: 10.3390/su10051528

[3] Chan C Jie L Jian H. Spatial–temporal patterns of population aging in rural China. Int J Environ Res Public Health. (2022) 19:15631. doi: 10.3390/IJERPH192315631 36497704 PMC9740567

[4] Shiode N Morita M Shiode S . Urban and rural geographies of aging: a local spatial correlation analysis of aging population measures. Urban Geogr. (2014) 35:608–28. doi: 10.1080/02723638.2014.905256

[5] Kim S Lee Y Oh H. Estimation of gridded population with spatial downscaling in South Korea. Sustainability. (2025) 17:1511. doi: 10.3390/SU17041511

[6] Zhang Y Luo H Xie J Meng X Ye C. The influence and prediction of built environment on the subjective well-being of the older adult based on random forest: evidence from Guangzhou, China. Land. (2023) 12:1940. doi: 10.3390/land12101940

[7] Tiebout CM. A pure theory of local expenditures. J Polit Econ. (1956) 64:416–24. doi: 10.1086/257839

[8] Talen E. The social equity of urban service distribution: an exploration of park access in Pueblo, Colorado, and Macon, Georgia. Urban Geogr. (1997) 18:521–41. doi: 10.2747/0272-3638.18.6.521

[9] Ujikawa T. Convenience stores as care infrastructure for older adults: the crisis of care in Tokyo. Int J Urban Reg Res. (2024) 49:183–203. doi: 10.1111/1468-2427.13292

[10] Zhang C Cao X Cheng J Gao Y De Vos J. Exploring the temporal variations in accessibility to health services for older adults in greater London. J Transp Health. (2022) 24:101334. doi: 10.1016/J.JTH.2022.101334

[11] Wong S Ponder CS Melix B. Spatial and racial COVID-19 disparities in U.S. nursing homes. Soc Sci Med. (2023) 325:115894. doi: 10.1016/j.socscimed.2023.115894, 37060641 PMC10080861

[12] Li Y Liu X. Effects of spatial accessibility of community health services on the activities of daily living among older adults in China: a propensity score matching study. Front Public Health. (2024) 12:121335712. doi: 10.3389/fpubh.2024.1335712, 38932781 PMC11199788

[13] Hao Z. Spatial matching and policy-planning evaluation of urban older adult care facilities based on multi-agent simulation: evidence from Shanghai, China. Sustainability. (2022) 14:16183. doi: 10.3390/SU142316183

[14] Li Y Qi W Liu S. Offsite pension or offspring caregiving: spatial differentiation and determinants of older adult migration motivations in China. Appl Spat Anal Policy. (2025) 18:123. doi: 10.1007/s12061-025-09733-8

[15] Samantha B Maxwell H. The 'double risk' of aging: examining vulnerability and (un)supportive built environments in Canadian cities. Can J Aging. (2023):11–5. doi: 10.1017/S0714980823000429 37665016

[16] Hu H Shao H Li Y . GIS-based analysis of older adult care facility distribution and supply–demand coordination in the Yangtze River Delta. Land. (2025) 14:723. doi: 10.3390/LAND14040723

[17] Zhang L Ren H Li C. Study on the development characteristics and spatial and temporal patterns of population ageing in 31 central cities in China. Front Public Health. (2024) 12:1341455. doi: 10.3389/FPUBH.2024.1341455 38699420 PMC11063271

[18] Xiaoran H Pixin G Marcus W . Research on spatial distribution characteristics and influencing factors of pension resources in Shanghai community-life circle. ISPRS Int J Geo Inf. (2022) 11:518. doi: 10.3390/IJGI11100518

[19] Sun X Cheng Y Tao Z. Spatial accessibility and equity of residential care facilities in Beijing from 2010 to 2020. Health Place. (2024) 86:103219. doi: 10.1016/J.HEALTHPLACE.2024.103219 38467103

[20] Zhang L Wei L Zhang W Fang Y. Bridging the gap: coordinating equity and efficiency in older people care resource allocation in China. BMC Geriatr. (2024) 24:165. doi: 10.1186/s12877-024-04696-w, 38365604 PMC10874015

[21] Sun J Huang J Jiang X Song X Zhang N. Spatiotemporal evolution and influencing factors of population aging in the triangle of Central China at multiple scales. Sustainability. (2025) 17:6549. doi: 10.3390/su17146549

[22] Xiangyang B Mo L. Equity versus efficiency: a spatial analysis of residential aged care resources in Beijing. Chin J Sociol. (2023) 9:127–58. doi: 10.3390/SU17146549

[23] Chen Y Bouferguene A Shirgaokar M Al-Hussein M. Spatial analysis framework for age-restricted communities integrating spatial distribution and accessibility evaluation. J Urban Plan Dev. (2020) 146:04019021. doi: 10.1061/(asce)up.1943-5444.0000537

[24] Li B Long J Li Y. Geospatial analysis of healthcare and older adult care institutions in Wuhan: a multimethod approach to assessing spatial equity. Front Public Health. (2025) 13:1580630. doi: 10.3389/fpubh.2025.1580630, 40837959 PMC12363478

[25] Hai L Cui Y Zhang J Wang R. The dynamic equilibrium between population aging and the allocation of older adult medical care resources in the Yellow River Basin. BMC Public Health. (2025) 25:2510. doi: 10.1186/s12889-025-22984-x, 40684144 PMC12275301

[26] Edmonds L Delamater P. Evaluating spatial access to hospitals for aging populations in legacy cities: a case study of Baltimore, Maryland. GeoJournal. (2025) 90:140. doi: 10.1007/s10708-025-11387-5

[27] Zhang Y Zhu J Li F Wang Y. Site selection of older adult care facilities based on multi-source spatial big data and integrated learning. ISPRS Int J Geo Inf. (2024) 13:451. doi: 10.3390/ijgi13120451

[28] Li M Peng P Ao Y . Equity in public decision-making: a dynamic comparative study of urban–rural older adult care institution resource allocation in China. Humanit Soc Sci Commun. (2024) 11:1526. doi: 10.1057/S41599-024-04041-X

[29] Wang C Geng X. Assessing the spatial equity of the aged care institutions based on the improved potential model: a case study in Shanghai, China. Front Public Health. (2024):121428424. doi: 10.3389/FPUBH.2024.1428424 PMC11390641 39267650

[30] Wei L Fang Y Zhang L. Identifying accessibility and equity defects of older adult care services in developing countries: insights from Xiamen City. J Nurs Manag. (2024) 2024:9340456. doi: 10.1155/2024/9340456, 40224830 PMC11919240

[31] Liu Q Zhao LS Li FY. Has the matching between urban population aging and older adult care facilities achieved coupling coordination?—an empirical analysis based on spatiotemporal evolution and multifactor interaction mechanisms. Front Public Health. (2025):131644849. doi: 10.1016/J.HABITATINT.2023.102828 PMC12321516 40766031

[32] Jin M Deng Q Wang S Wei L. Equity evaluation of older adult-care institutions based on Ga2SFCA: the case study of Jinan, China. Sustainability. (2023) 15:16943. doi: 10.3390/SU152416943

[33] Striessnig E Gao J O’Neill CB Jiang L. Empirically based spatial projections of US population age structure consistent with the shared socioeconomic pathways. Environ Res Lett. (2019) 14:114038. doi: 10.1088/1748-9326/ab4a3a

[34] Dao DHT Dao AK Salerno BG. Uncovering spatial patterns of residential settlements, segregation, and vulnerability of urban seniors using geospatial analytics and modeling techniques. Urban Sci. (2024) 8:81. doi: 10.3390/URBANSCI8030081

[35] Yin X Cui J Wu Y Cui M Li K Guo H. Spatial–temporal evolution and associated factors of older adult care institutions in Shanghai. Front Public Health. (2025):131598394. doi: 10.3389/FPUBH.2025.1598394 PMC12267019 40678644

[36] Xu Y Chen S Wang Z Liu B Wang L. Multi-scale dynamics and spatial consistency of economy and population based on NPP/VIIRS nighttime light data and population imagery: a case study of the Yangtze River Delta. Remote Sens. (2024) 16:2806. doi: 10.3390/RS16152806

[37] Lianhua L Yanling W Shiqi L Zexian C. Equilibrium study of logistics demand and logistics resource allocation in Guangdong Province. Sci Rep. (2025) 15:3805. doi: 10.1038/S41598-025-88504-4 39885327 PMC11782670

[38] Xiang Y He S. Healthcare in cumulatively caused migration: Hong Kong residents’ perceived mainland healthcare quality and migration intentions in the Greater Bay Area, China. Habitat Int. (2023) 136. doi: 10.1016/J.HABITATINT.2023.102828

[39] Weidong W. China’s health service collaboration in the Guangdong-Hong Kong-Macao Greater Bay Area: barriers and next steps. Front Public Health. (2025) 13:1442328. doi: 10.3389/fpubh.2025.1442328 40496450 PMC12149214

[40] Lai W Lin D Li Z Peng Y Zhou W Feng T. Grid-level assessment on spatial equity in access to urban public facilities by vulnerable groups based on the multi-source data. Habitat Int. (2025) 161:103423. doi: 10.1016/j.habitatint.2025.103423

[41] Feng H Tang X Zou C. Optimizing the layout of service facilities for older people based on POI data and machine learning: Guangzhou City as an example. Land. (2024) 13. doi: 10.3390/land13050700

[42] Zhang X Shi J Chao M Yin J. Study on the differences and influencing factors of spatial distribution of population aging at township scale: a case study of township research units in Anshun City, China. Front Public Health. (2024) 12:1351395. doi: 10.3389/FPUBH.2024.1351395 38605876 PMC11008717

[43] Ke Z Hao S Xiangyu L. Aging population spatial distribution discrepancy and impacting factor. Sustainability. (2022) 14:9528. doi: 10.3390/SU14159528

[44] Dunyi G Lei L Zenglin H. Spatial-temporal variation of population aging: a case study of China’s Liaoning Province. Complexity. (2020). doi: 10.1155/2020/5436061

[45] Fangzhu Z Fulong W Weikai W Chun Y Yin MC Juan W . Cross-border ageing in China’s Greater Bay Area in the digital age: a comparative study of mobile application adoption by Hong Kong older migrants and local older adults in Shenzhen. Trans Plann. Urban Res. (2023) 2:149–66. doi: 10.1177/27541223221150653

[46] Song S Guilin L. Spatial matching relationship between health tourism destinations and population aging in the Yangtze River Delta urban agglomeration. Environmental research. Communications. (2023) 5. doi: 10.1088/2515-7620/acf3d4

[47] Huanhuan Z Lin P Yiji L Huiming J Qian W Xin L . Spatial accessibility assessment of prehospital EMS with a focus on the older adult population: a case study in Ningbo, China. Int J Environ Res Public Health. (2021) 18:9964. doi: 10.3390/IJERPH18199964 34639264 PMC8508414

[48] Yang M Rosenberg WM Li J. Spatial variability of health inequalities of older people in China and related health factors. Int J Environ Res Public Health. (2020) 17:1739. doi: 10.3390/ijerph17051739 32155968 PMC7084825

